# Combined Skin and Muscle DNA Priming Provides Enhanced Humoral Responses to a Human Immunodeficency Virus Type 1 Clade C Envelope Vaccine

**DOI:** 10.1089/hum.2018.075

**Published:** 2018-10-17

**Authors:** Hannah Mary Cheeseman, Suzanne Day, Leon Robert McFarlane, Sue Fleck, Aleisha Miller, Tom Cole, Nelson Sousa-Santos, Alethea Cope, Deniz Cizmeci, Monica Tolazzi, Edith Hwekwete, Drew Hannaman, Sven Kratochvil, Paul Francis McKay, Amy W. Chung, Stephen J. Kent, Adrian Cook, Gabriella Scarlatti, Sonya Abraham, Behazine Combadiere, Sheena McCormack, David John Lewis, Robin John Shattock

**Affiliations:** ^1^ Department of Medicine, Section of Virology, Group of Mucosal Infection and Immunity, Imperial College London, London, United Kingdom.; ^2^ Medical Research Council Clinical Trials Unit at UCL, University College London, London, United Kingdom.; ^3^ Imperial Clinical Research Facility, Hammersmith Hospital, Imperial College Healthcare NHS Trust, United Kingdom.; ^4^ Viral Evolution and Transmission Unit, Division of Immunology, Transplant and Infectious Diseases, San Raffaele Scientific Institute, Milan, Italy.; ^5^ Ichor Medical Systems, Inc., San Diego, California.; ^6^ Department of Microbiology and Immunology, Peter Doherty Institute for Infection and Immunity, University of Melbourne, Melbourne, Australia.; ^7^ ARC Centre of Excellence in Convergent Bio-Nano Science and Technology, University of Melbourne, Melbourne, Australia.; ^8^ Melbourne Sexual Health Centre, Department of Infectious Diseases, Alfred Health, Central Clinical School, Monash University, Melbourne, Australia.; ^9^ Sorbonne Universités, UPMC Univ Paris 06, INSERM, U1135, CNRS, ERL 8255, Centre d'Immunologie et des Maladies Infectieuses (CIMI-Paris), Paris, France.

**Keywords:** HIV, DNA vaccine, electroporation, antibody, gp140

## Abstract

Intradermal (i.d.) and intramuscular (i.m.) injections when administered with or without electroporation (EP) have the potential to tailor the immune response to DNA vaccination. This Phase I randomized controlled clinical trial in human immunodeficiency virus type 1–negative volunteers investigated whether the site and mode of DNA vaccination influences the quality of induced cellular and humoral immune responses following the DNA priming phase and subsequent protein boost with recombinant clade C CN54 gp140. A strategy of concurrent i.d. and i.m. DNA immunizations administered with or without EP was adopted. Subtle differences were observed in the shaping of vaccine-induced virus-specific CD4+ and CD8+ T cell–mediated immune responses between groups receiving: i.d._EP_ + i.m., i.d. + i.m._EP_, and i.d._EP_ + i.m._EP_ regimens. The DNA priming phase induced 100% seroconversion in all of the groups. A single, non-adjuvanted protein boost induced a rapid and profound increase in binding antibodies in all groups, with a trend for higher responses in i.d._EP_ + i.m._EP_. The magnitude of antigen-specific binding immunoglobulin G correlated with neutralization of closely matched clade C 93MW965 virus and Fc-dimer receptor binding (FcγRIIa and FcγRIIIa). These results offer new perspectives on the use of combined skin and muscle DNA immunization in priming humoral and cellular responses to recombinant protein.

## Introduction

DNA-based vaccination is an attractive mode of vaccine delivery, particularly against viral infections. DNA vaccines utilize the host for *in vivo* biosynthesis of transgene products,^1^ hence imitating infectious pathways, and through host cell post-translational modifications, the transgene products more accurately represent the conformation of naturally expressed viral antigens.^2^ A lack of anti-vector immunity provides the opportunity for serial immunizations with multiple DNA derived immunogens. DNA vaccination is typically used as a component of heterologous prime-boost strategies. In the context of generating humoral responses, prime-boost vaccination is generally thought to induce memory T-cell responses^3^ able to boost subsequent T cell–dependent antibody responses to recombinant antigens.^4^ However, the extent to which DNA vaccination is able to prime antigen-specific B cells directly, influencing their antigen specificity, is less clear and likely dependent on the intrinsic antigenicity of the vaccinating immunogen. In this respect, the human immunodeficiency virus type 1 (HIV-1) envelope glycoprotein presents a particular challenge, known to be poorly immunogenic due in part to the very high density of glycans that restrict antibody recognition of the underlying protein.^5,6^ Indeed, with two notable exceptions,^7,8^ the majority of clinical vaccine studies using injected naked plasmid DNA encoding HIV-1 envelope glycoproteins have failed to induce detectable antibody responses.^9^

Nevertheless, over recent years, the immunogenicity of DNA vaccines has been significantly enhanced through the use of promoter selection and codon optimization.^1^ Furthermore, the delivery of DNA in association with electroporation (EP) has been shown to increase gene expression and vaccine-induced responses dramatically.^10–13^ EP generates an electric field at the vaccine site, creating temporary cell membrane instability, thereby facilitating increased uptake of DNA. Importantly, the inflammation associated with EP is also thought to enhance antigen-presenting cell (APC) recruitment.^14,15^ In recent Phase I HIV-1 prophylactic vaccine trials, DNA vaccination with EP has been shown to improve cell-mediated immunity (CMI), while its impact on antibody induction was minimal or below the level of detection.^16,17^ The route of vaccination is also thought to have profound effects on prevailing immune responses. Most DNA vaccinations are delivered via the intramuscular (i.m.) route.^16^ However, the low number of APC within muscle tissues may be a rate-limiting factor in inducing robust humoral responses.^1,18,19^ By contrast, the skin has relatively high numbers of resident APC able to migrate to the draining lymph nodes via lymphatic drainage where they preferentially interact with CD4+ T follicular helper (Tfh) cells, inducing naive B cells to make antibody.^20^ However, the volumes that can be delivered via the intradermal (i.d.) route are much smaller than can be delivered i.m., providing a practical constraint when considering this route. Few clinical studies have directly compared the performance of the two routes for delivery of DNA, but preliminary data suggest i.d. administration to be dose sparing and superior with or without EP.^21,22^ These findings are supported by small-animal data, suggesting that greater polyfunctional CD4+ T-cell responses are seen after i.d. administration of a DNA vaccine, even at 20% of the dose, when the same vaccine is administered i.m.^23^ Furthermore, comparative studies of i.d. and i.m. delivery followed by *in vivo* EP of SIV Env DNA in macaques suggested that i.d._EP_ induced higher cross-reactive antibody responses than i.m._EP_, while cell-mediated response induced by i.m._EP_ were 10-fold higher than those induced by i.d._EP_.^24^ The predictive nature of these studies for human immunogenicity is unclear.

This study sought to develop a DNA priming strategy capable of reproducibly inducing detectable B-cell responses, evident before boosting with recombinant protein. This has important relevance to current multicomponent approaches for inducing protective HIV-1 antibody responses, where a series of immunogens maybe required to focus humoral immune responses to recognize rarely induced broadly neutralizing epitopes.^6^ Given the high cost of recombinant protein manufacture, the potential use of a series of DNA priming immunizations prior to boosting of responses with a single recombinant protein would provide an attractive and cost-effective strategy for evaluating multicomponent vaccines.

It was hypothesized that combining the i.d. route, which allows the vaccine to reach a site with a plentiful and diverse number of APCs,^25^ with concurrent i.m. vaccination, which allows delivery of a higher concentration of DNA, might result in enhanced immune responses to the *in vivo*-expressed plasmid DNA vaccine transgenes. Previous preclinical murine studies supported this hypothesis, but the extent to which this would translate to human studies was unclear.^26^ More recently, in a human clinical study using a T cell–based vaccine, it was observed that combined i.d. and i.m. vaccination differentially impacted the quality of effector T-cell functions.^27^ This study sought to optimize this regimen to augment vaccine-induced humoral immunity. As a first phase in developing this approach, a single DNA plasmid vector expressing *CN54 gp140* was used to deliver *HIV-1 Env* as a priming vaccine immunogen. The impact of EP on concurrent i.d. and i.m. vaccinations to prime humoral and cellular responses was evaluated prior to boosting with a model recombinant HIV-1 vaccine antigen, CN54-gp140. The study deliberately did not use an adjuvant for the protein boost in order to differentiate clearly the impact of the different components of the concurrent regimens. While humoral antibody responses were the principal focus of this study, gp140-specific cellular responses were also evaluated, as CD4 T-cell help is critical not only for the development of cytotoxic CD8 responses but also for the development of Env-specific functional antibody responses. The results presented here demonstrate that concurrent i.d./i.m. vaccination is a highly effective activator of both cellular and humoral responses. Of particular note is that the i.d._EP_ + i.m._EP_ combination generated both T- and B-cell responses that were dramatically amplified by a homologous protein boost in the absence of additional adjuvantation.

To the best of the authors' knowledge, this is the first clinical study to evaluate concurrent i.d./i.m. plasmid DNA vaccination as part of a DNA prime-protein boost strategy.

## Methods

### Trial design

**Figure 1.**
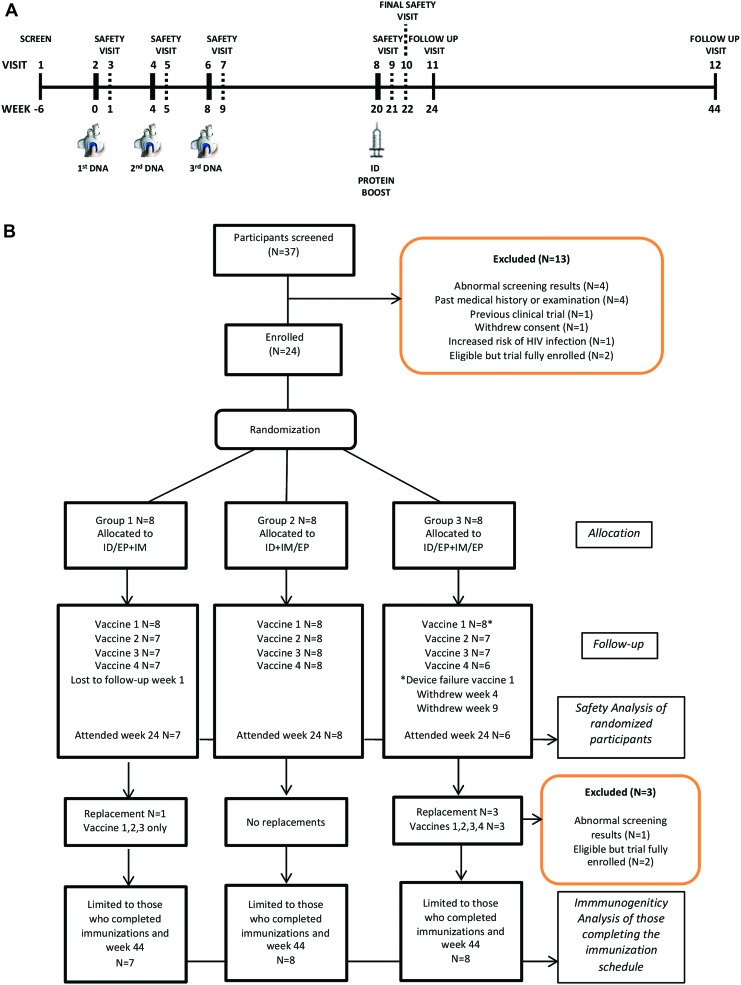
**(A)** Trial schematic. Participants attended a total of 12 visits, with DNA immunizations given at week 0 (visit 2), week 4 (visit 4), and week 8 (visit 6) followed by an intradermal (i.d.) booster injection of recombinant CN54gp140 at week 20 (visit 8). Blood was taken from participants at each visit, and mucosal samples were taken at weeks 0, 20, and 22 (visits 2, 8, and 10, respectively). *Thick solid lines* indicate an immunization visit (weeks 0, 4, 8, and 20). *Dotted lines* indicate a safety visit (weeks 1, 5, 9, 21, and 22). *Thin solid lines* indicate a screen or follow-up visit (weeks 6, 24, and 44). **(B)** CONSORT flow diagram. Participants were randomized into one of three groups. The numbers of participants enrolled, randomized, followed up, and analyzed are shown for each treatment group. Color images available online at www.liebertpub.com/hum

CUTHIVAC002 was a randomized, open label, Phase I HIV vaccine trial in healthy volunteers aged 18–50 years. The target sample size was 24 participants who completed all immunizations. Participants were randomized into one of three methods (i.d._EP_ + i.m., i.d. + i.m._EP_, and i.d._EP_ + i.m._EP_) of delivering DNA-C *CN54ENV* at weeks 0, 4, and 8 and subsequently boosted with recombinant CN54gp140 at week 20 (Fig. 1A). The randomization method was block randomization using a computer-generated algorithm, stratified on sex, and implemented through the Cactus electronic data capture system on the day of enrolment and first immunization after confirmation of eligibility. Group 1 (i.d._EP_ + i.m.) received i.d. immunization with EP combined with i.m. immunization. Group 2 (i.d. + i.m._EP_) received i.d. immunization combined with i.m. immunization with EP. Group 3 (i.d._EP_ + i.m._EP_) received i.d. immunization with EP combined with i.m. immunization with EP. Protein boosts were administered by i.d. injection. Each volunteer was invited to attend 11 visits from screening through to 4 weeks after the final immunization, with an additional week 44 visit to assess durability of induced humoral responses. Those who did not complete the immunization schedule were replaced without randomization.

Clinical trial authorization was granted by the British Medicines and Healthcare products Regulatory Agency. Favorable ethical approval opinion was given by the London Riverside Research Ethics Committee, operating within the Research Ethics Service of the British Health Research Authority. The trial was registered with the European Union Drug Regulating Authorities for Clinical Trials (EudraCT) database and assigned the EudraCT No. 2015-001023-23 and was registered with ClinicalTrials.gov under protocol ID NCT02589795 (date of registration October 28, 2015). The trial was performed at the NIHR Trust Imperial Clinical Research Facility, Hammersmith Hospital, Imperial College Healthcare NHS Trust (London, United Kingdom).

### Study participants and eligibility criteria

Healthy adults aged between 18 and 50 years at low risk of HIV-1 infection and with no clinically significant medical history or disorder that presented the potential for risk of influencing the results or impairing the participant's ability to participate in the study were enrolled. All participants provided written informed consent.

### Interventions

Three different methods of delivering a DNA vaccine (DNA-C *CN54ENV*) via combined i.m. and i.d. routes with and without EP were explored, all followed by a single recombinant HIV CN54gp140 administered by i.d. injection without EP. Staff were trained in the injection and EP techniques as part of the trial specific training.

### DNA-C *CN54ENV*

The codon-optimized plasmid (DNA-C *CN54ENV*) encodes the HIV-1 clade C gp140 (env) derived from 97CN54 and was manufactured in accordance with Good Manufacturing Practice (GMP) by Ajinomoto Althea Technologies (San Diego, CA). The plasmid was formulated in phosphate-buffered saline (PBS), pH 7.2, and supplied as a sterile solution for injection.

### Recombinant CN54 gp140

CN54gp140 is a recombinant gp140 derived from the HIV-1 97CN54 Env coding sequence manufactured in accordance with GMP by Polymun Scientific Immunbiologische Forschung GmbH (Klosterneuburg, Austria). The protein comprises a sequence of 670 amino acids and has previously been shown to be immunogenic in humans.^28,29^ The protein is heavily glycosylated and has a mass of approximately 140 kDa, as determined by sodium dodecyl sulfate polyacrylamide gel electrophoresis and size-exclusion chromatography. Furthermore, the CN54gp140 secreted by CHO cells is oligomeric, and following purification is essentially trimeric, with a projected mass of 420 kDa.

### Intradermal DNA administration

DNA-C *CN54ENV* was administered in the absence of EP as a 1 × 0.15 mL (0.6 mg) i.d. injection via a small needle (29 gauge), inserted into the dermis overlying the deltoid muscle of the non-dominant arm. An i.d. adapter (West Pharmaceutical Services, Inc., Exton, PA) was used to facilitate the injection and to ensure consistency of administration. The “bleb” or weal created was used as an indicator of successful injection into the i.d. layer. Intradermal DNA injection with EP was administered via needle-free jet injection (Medi-jector Vision; Antares Pharma, Ewing Township, NJ) within the handheld TDS-ID integrated applicator (TriGrid™ Delivery System; Ichor Medical Systems, San Diego, CA) as a 1 × 0.15 mL (0.6 mg) injection into the dermis overlying the deltoid muscle of the non-dominant arm. Intradermal administration was immediately followed by the application of electrical stimulation. The electrical field was applied at an amplitude of 330 V/cm of electrode spacing for a duration of 40 ms, within a 400 ms interval, resulting in brief muscle contractions.

### Intramuscular DNA administration

DNA-C *CN54ENV* was administered as a 1 × 0.5 mL (2 mg) injection via a needle into the vastus lateralis muscle of the upper thigh. For i.m. DNA injection with EP, the disposable EP cartridge was loaded with the 1 × 0.5 mL (2 mg) DNA vaccine and then adjusted to one of three depth settings, corresponding to predefined ranges in skin-fold thickness. The cartridge was loaded into the EP device and applied to the vastus lateralis muscle, followed immediately by the application of electrical stimulation and brief muscle contractions (TriGrid™ Delivery System; Ichor Medical Systems). The spacing of the TriGrid™ electrode array was 6 mm in a diamond-shaped configuration, and the electrical field was applied at an amplitude of 250 V/cm of electrode spacing for a 40 ms total duration applied as three pulses over a 400 ms interval, resulting in brief muscle contractions.

### Intradermal protein boost injection

The CN54gp140 vaccine (0.5 mg/mL) as provided by the manufacturer was administered as 1 × 0.10 mL (50 μg) injection, as described above for the i.d. DNA injection without EP.

### Objectives and primary endpoint measures

The main objective was to investigate the safety and immunogenicity of the three different methods of administering *CN54ENV C* clade DNA vaccine (i.d._EP_ + i.m., i.d. + i.m._EP_, and i.d._EP_ + i.m._EP_) followed by a single i.d. boost with recombinant CN54gp140 protein (Fig. 1A). The primary safety endpoint was a Grade 3 or above solicited local, systemic, or laboratory adverse event, or any grade of other adverse event leading to a clinical decision to discontinue immunizations or with onset within 7 days of immunization. The primary immunogenicity endpoint was the magnitude of antigen-specific systemic immunoglobulin G (IgG) antibody binding responses (ng/mL) to CN54gp140 measured by binding enzyme-linked immunosorbent assay (ELISA) using serum collected 2 weeks after the final immunization.

### Reactogenicity and safety

Participants were monitored in the clinic for at least 60 min after each vaccination, and contacted by telephone a day later. They completed diary cards for 7 days, and returned for review within 7 days when they were asked about other adverse events and diary cards were checked. The grade of solicited local and systemic adverse events starting within 7 days of immunization, and other adverse events were recorded in the case record form and diary card and entered in the study database for randomized participants. Systemic safety was also assessed through routine laboratory parameters (full blood count and differential, renal, and liver biochemistry), and reported in the electronic database. Adverse events were graded using modified U.S. Food and Drug Administration and Division of AIDS criteria.

### Collection and processing of mucosal secretions

Cervicovaginal secretions were collected from female volunteers using an Instead Softcup, a commercially available, self-inserted menstrual cup made of polyethylene. The Softcup was inserted into the upper vagina, left in place for at least 1 h, removed, and stored in a 50 mL Falcon tube at −80°C. Mucosal samples were collected at weeks 0, 20, and 22. Prior to processing the mucosal samples, the Softcups containing cervicovaginal secretions were removed from storage at −80°C and thawed overnight at 2–8°C. Following centrifugation at 400 *g* for 15 min at 4°C, an equal volume of extraction buffer (1 × protease inhibitor cocktail; Calbiochem, San Diego, CA), 0.02% NaN_3_ (Sigma–Aldrich, St. Louis, MO), and 0.25 M NaCl (BDH) in sterile PBS (Gibco, Waltham, MA)) was added to the secretion samples (1:1 dilution). Samples were then aliquoted and stored at −80°C until analysis.

Rectal secretions were collected using a PVA sponge, pre-moistened with 50 μL saline and stored at −80°C. Sponges were removed from storage at −80°C, thawed on wet ice, and placed into a 50 mL tube containing a sterile 70 μm Falcon filter unit. Extraction buffer (500 μL) was added before centrifugation at 400 *g* for 10 min at 4°C. To remove debris, the eluate was transferred into the top chamber of a Spin–X tube, centrifuged at 16,000 g for 15 min at 4°C, aliquoted, and stored at −80°C until analysis.

### Peripheral blood mononuclear cell sample preparation

Peripheral blood mononuclear cells (PBMC) were isolated using density gradient separation from heparinized whole blood. Cells were processed within 4 h of collection and stored at 1 × 10^7^ cells/mL in freezing media (10% dimethyl sulfoxide, 90% fetal bovine serum; Sigma–Aldrich) at −80°C before storage in vapor phase liquid nitrogen prior to analysis.

### Memory B-cell enzyme-linked immunospot

B-cell enzyme-linked immunospot (ELISpot) was used to assess the magnitude of CN54gp140 antigen-specific memory B cells (mBC) responses. In brief, frozen PBMC were thawed and re-suspended at 1 × 10^6^ cells/mL in stimulation media composed of 5 ng/mL interleukin (IL)-2 (Roche, Welwyn Garden City, United Kingdom) and 0.5 μg/mL R848 (Invivogen, San Diego, CA) before being cultured for 4 days at 37°C, 5% CO_2_. Sterile 96-well ELISpot plates (Millipore, Billerica, MA) were pre-wet with 15 μL/well 70% EtOH for 1 min before washing with Dulbecco's PBS. Wells were coated overnight at 4°C with 5 μg/mL CN54gp140-antigen, 15 μg/mL of the capture antibody MT91/145 (Mabtech, Stockholm, Sweden), or PBS only. Plates were blocked with RPMI supplemented with 10% heat-inactivated fetal calf serum (FCS; R10) for 1–2 h before the addition of 10,000 cells/well for total IgG and 200,000 cells/well for antigen-specific responses. Each condition was tested in triplicate, and plates were incubated for 6 h at 37°C, 5% CO_2_. Biotinylated detection antibody MT78/145 (Mabtech) was added at 1 μg/mL in PBS/0.5% FCS and incubated overnight at 4°C. The next day, streptavidin-horseradish peroxidase (HRP; Mabtech) diluted 1:1000 in PBS/0.5% FCS was added and incubated for 1 h at room temperature. AEC substrate solution (BD Biosciences, San Diego, CA) was added and incubated for 5–10 min before stopping the spot development with tap water. Spot-forming units (SFU) were counted using an automated AID ELISpot reader (Autoimmun Diagnostika GmbH, Straßberg, Germany).

### Interferon gamma ELISpot

Interferon gamma (IFN-γ) ELISpot assays were performed using frozen isolated PBMC stimulated with peptide pools matched to the vaccine at sampling weeks, 0, 20 and 22. In brief, frozen PBMC were rested overnight at a concentration of 2.5 × 10^6^ PBMC/mL and re-suspended to a final concentration of 4 × 10^6^ viable PBMC/mL (4 × 10^5^ PBMC/mL for positive control phytohemagglutinin [PHA] wells). Pre-coated IFN-γ ELISpot 96-well plates (Mabtech) were washed with sterile PBS and blocked with R10 media before the addition of 50 μL cells/well in triplicate with 50 μL media only, stimulation media containing vaccine-specific peptide pools Env 1, 2, and 3 (15-mers overlapping by 11 covering the entire sequence of CN54gp140 clas C HIV-1) and two positive controls PHA; Sigma–Aldrich; and cytomegalovirus/Epstein Barr virus/influenza virus/tetanus [CEFT]; (thinkpeptides, Oxford, United Kingdom) at a final concentration of 2.5 μg/mL. Plates were incubated for 16–24 h at 37°C, 5% CO_2_. Plates were then washed and incubated for 2 h at room temperature with 1 μg/mL mouse-anti human IFN-γ (Mabtech). The signal was amplified with 1 h incubation with streptavidin-ALP solution (Mabtech), then developed with substrate, 5-bromo-4-chloro-3′-indolyphosphate and nitro-blue tetrazolium (BCIP/NBT plus; Mabtech) for 5–7 min. The reaction was stopped by washing with tap water and allowed to dry overnight in the dark. Plates were read with an automated AID iSPOT ELISpot plate reader (Autoimmun Diagnostika GmbH). The number of SFU/10^6^ PBMC was calculated as the mean count subtracting the background count. Pass/fail criteria for the assay was dependent on the mean of the negative wells <100 SFU/10^6^ PBMC, and the PHA-positive response was >500 SFU/10^6^ PBMC.

### Intracellular cytokine analysis

T-cell responses were evaluated by intracellular cytokine staining (ICS) with a panel for CD8+ and CD4+ antigen-specific responses. In brief, cryopreserved PBMC were thawed and rested overnight in R10 media at 37°C, 5% CO_2_. 1 × 10^6^ viable PBMC were stimulated with 2.5 μg/mL of each vaccine-matched peptide pools (*Env 1, 2,* and *3*) plus 1 μg/mL CD28/49d (BD Biosciences) for 6 h at 37C. Phorbol 12-myristate 13-acetate/ionomycin (Sigma–Aldrich), and CEFT were used as positive controls and R10 media with 1.5% DMSO was used as a negative control. Two hours into the stimulation, Brefeldin A (eBioscience, San Diego, CA) was added. Cells were stained with fixable viability dye eFluor780 (eBioscience), CD3 BV650 (Clone OKT3), CD4 PerCP-Cy5.5 (RPA-T4), CD8 AF700 (RPA-T8), IFN-γ AF488 (4S.B3), TNF-α PE-Cy7 (MAb11), IL-2 BV510 (MQ1-17H12), CD154 BV421 (24–31) (BioLegend, San Diego, CA), Granzyme B AF647 (GB11; BD Biosciences), and Perforin PE (dG9; eBioscienceCA) to assess CD4/CD8-specific responses. Cells were fixed and stored at 2–8°C before analysis on a FortessaLSRII (Becton Dickinson, Franklin Lakes, NJ) equipped with 50 mW 405 nm, 50 mW 488 nm, 50 mW 561 nm, and 20 mW 633 nm lasers and a ND1.0 filter in front of the FSC photodiode. Acquisition was set to record 50,000 live CD3+ lymphocytes after dead cell and doublet exclusion (FSC-A/W, SSC-A/W gating). Analysis was performed using FlowJo v10 software (Treestar, Ashland, OR), PESTLE and SPICE software (courtesy of Mario Roederer, Vaccine Research Center, National Institutes of Allergy and Infectious Diseases). Data were expressed as the percentage of total CD4+ or CD8+ cells. Background responses in negative controls were subtracted from the stimulated samples. Responders were defined as having three times the background and a percentage staining >0.05% after subtraction of the baseline (prior to vaccination) result.

### HIV-specific binding antibodies

Antigen-specific gp140 binding antibodies were measured using standardized ELISA platforms. In serum samples, antigen-specific IgA, IgG, and IgG subclasses were measured. In brief, 96-well high binding plates (Griener, Kremsmünster, Austria) were coated with recombinant CN54gp140 (Polymun Scientific) at 1 μg/mL in PBS for 1 h. As reference material, standard immunoglobulins (Sigma–Aldrich) were captured with anti-human kappa and lambda light chain specific mouse antibodies (Southern Biotech, Birmingham, AL). After blocking with assay buffer (1% bovine serum albumin; Sigma–Aldrich; 0.05% Tween Thermo Fisher Scientific, Pittsburgh, PA), samples were initially screened at 1:100 dilution (then titrated to optimal dilutions) on antigen-coated wells, and serial dilutions of immunoglobulin standards were added to the kappa/lambda capture antibody coated wells and incubated for 1 h at 37°C. Secondary antibody HRP-conjugated anti-human IgG was added at 1:20,000 dilution and incubated for 1 h at 37°C. Plates were developed with SureBlue TMB substrate (KPL, Insight Biotechnology, London, United Kingdom). The reaction was stopped after 5 min by adding TMB stop solution (KPL, Insight Biotechnology), and the absorbance was read at 450 nm on a VersaMax 96-well microplate reader (Molecular Devices, Sunnyvale, CA). The ELISA data are expressed as positive if the blank-subtracted OD 450 nm was above the predetermined cutoff of OD 0.2 nm and values are on the linear range of the curve. To ensure assay sensitivity, a positive control composed of positive pooled plasma samples was used. Analyses of the data were performed using SoftMax Pro GxP software v6.5 (Molecular Devices).

IgG subclasses 1–4 were investigated for serum samples only. The protocol was followed as above with the use of IgG1–4 standards (Abcam, Cambridge, United Kingdom and Sigma–Aldrich) and mouse anti-human IgG1 HRP (Acris, Rockville, MD), mouse anti-human IgG3 HRP (Southern Biotech), or mouse anti-human IgG4 HRP (BD Biosciences). The IgG2 assay included a mouse anti-human IgG2-biotin (BD Biosciences) step followed by 1:200 streptavidin-HRP (RD Systems, Minneapolis, MN), as previously described.^28^

Antigen-specific IgA/G antibody responses were measured in the mucosal compartments (vaginal and rectal) at weeks 0, 20, and 22 using a supersensitive polyHRP ELISA. In addition, any serum samples determined to be below the limit of quantification in the conventional ELISA were retested using this assay. In brief, 96-well high binding plates (Nunc, Roskilde, Denmark) were coated overnight with antigen and capture antibody (as above) at 4°C. Casein buffer (Thermo Fisher Scientific, Waltham, MA) was used to block plates before addition of mucosal samples and standards (serially diluted 1:10) for 1 h at 37°C. Secondary antibody, biotin-conjugated anti-human IgG (Southern Biotech) was added and incubated for 1 h at 37°C before addition of streptavidin poly-HRP40 (Fitzgerald, Acton, MA) for 40 min at room temperature. Plates were developed and read as detailed for the conventional ELISA.

### Customized multiplex dimer assay

A customized multivariate multiplex assay was developed using a panel of gp140 antigens (Clade C: CN54, 1086; Clade A: UG37; Clade B: 9021, JRFL; Clade D: UG21; Clade F BR29 and M consensus NIH AIDS Reagents) coupled to magnetic fluorescent multiplex beads (Bio-Rad), as described previously.^30^ Biotinylated dimeric Fc-gamma-Receptors (FcγRIIa-H131, FcγRIIIa-V158) were produced as previously described,^31^ and kindly supplied by Drs. Bruce Wines and Mark Hogarth, Burnet Institute (Melbourne, Australia).

Coupled microspheres were premixed in multiplex assay buffer (PBS +0.1% BSA +0.05% Tween), creating a working mixture of 12.5 microspheres/bead type/μL. Using a black, clear-bottom 96-well plate, 40 μL of the working microsphere mixture (1,000 beads of each type/well) was added to 40 μL of 100 × diluted serum (diluted in PBS). The plate was covered and incubated overnight at 4°C on a plate shaker. The plate was washed three times with 200 μL of assay wash (PBS-1 × , 0.1% BSA, 0.5% Triton-100 × ) using a Bio-Plex Pro plate wash station (Bio-Rad). Antigen-specific antibody binding to dimeric FcγRs was detected by adding FcγRs followed by streptavidin PE at 1.0 μg/mL with 50 μg/well. After 2 h incubation at room temperature on a shaker, the plate was washed three times with 200 μL of assay wash, and microspheres were re-suspended in 100 μL of sheath fluid.

A Bio-Plex reader (Bio-Plex MAGPIX, Bio-Plex Manager 5.0; Bio-Rad) was used to detect the microspheres, and binding of PE was measured to calculate a median fluorescence intensity (MFI). Background signal, defined as the average MFI observed for each microsphere set when incubated with the PE-conjugated detection reagent in the absence of clinical antibody sample, was subtracted from the MFI for each sample.

### HIV-1 neutralization assay

Env-pseudotyped viruses (PSV) SF162 and MNec.3 (subtype B), 93MW965.26 (subtype C), and TH023.06 (CRF01-AE) were produced in HEK293T cells, titered, and used in TZM-bl assay to determine neutralizing antibody responses.^32^ Briefly, duplicates of six steps of threefold dilution, starting with 1:20 of each serum obtained at weeks 0 and 22, were incubated with viral supernatant (at relative luminescence units [RLU] between 148,000 and 272,000) for 1 h. Thereafter, 10^4^ TZM-bl cells were added, and plates were incubated for 48 h, when luciferase activity was measured by addition of Bright-Glo Luciferase assay system (Promega, Madison, WI). Neutralization titers were defined as the sample dilution at which RLU were reduced by 50% compared to virus control wells after subtraction of background RLU in control wells with only cells.

### Statistical methods

The primary safety outcome was expressed as a proportion of participants with 95% confidence interval, and groups were compared using Fisher's exact test. There was no pre-specified hypothesis on which to power the study. Immunological analyses were based on the per protocol (PP) population that received all vaccinations. All immunology data were performed observer blinded by use of a randomly generated laboratory identifier. Appropriate comparative statistics are annotated in the text. For the immunogenicity analysis, the primary end point was defined as 2 weeks after the fourth and final vaccine (week 20). Statistical analysis was carried out using Prism v7.04 (GraphPad Software, Inc., San Diego, CA) or R v3.3.2 (R Foundation, Vienna, Austria).

## Results

### Concurrent i.d. and i.m. DNA vaccination with and without EP and protein boost schedule was well tolerated in healthy HIV-negative adult volunteers

Study volunteers were recruited between August 2016 and February 2017. Twenty-eight were screened during the randomized period, with 24 deemed eligible and enrolled (Supplementary Table S1; Supplementary Data are available online at www.liebertpub.com/hum). Two participants (i.d._EP_ + i.m._EP_) withdrew consent, and one (i.d._EP_ + i.m.) was lost to follow-up during the immunization period. The TDS-ID device failed to deliver the first dose of vaccine in a fourth participant (CV024B) included in i.d._EP_ + i.m._EP_ (Fig. 1B). Seven volunteers were screened and four additional eligible participants enrolled to replace the individuals who did not complete their immunizations, matched on sex but not age. One (i.d._EP_ + i.m.) of the four replacement participants did not receive the CN54gp140 boost because, for personal reasons, he wished to minimize the longevity of vaccine-induced seroreactivity in HIV tests. A total of 28 volunteers received 103 of 112 planned doses of vaccines between August 30, 2016, and July 17, 2017, and 23 completed the immunization schedule. The majority of randomized participants were white British (71%) and male (75%) with a mean age of 27 years. The four participants recruited to replace those who did not complete their immunizations were similar, with a mean age of 33 years (Supplementary Tables S2 and S3).

All participants attended the safety visit within a week of their last immunization, and 25 attended the final safety visit at week 24. There were no adverse events that resulted in a clinical decision to discontinue vaccines among randomized and non-randomized participants. There was one serious adverse event: a road traffic accident that occurred 5 months after the last vaccine. There were two severe (grade 3) solicited events reported by randomized participants: arm discomfort after the third vaccination (i.d._EP_ + i.m.) and elevated aspartate transaminase after the first vaccination (i.d. + i.m._EP_). One of the non-randomized individuals (i.d._EP_ + i.m._EP_) reported two events after her third immunization: severe tiredness and severe general muscle aches. There were 23 non-solicited adverse events with onset within 7 days of immunization reported by randomized participants and two by non-randomized participants; these were mainly musculoskeletal or infection, and they were mild or moderate in severity.

Of the 365 events reported by the randomized participants, which includes the primary outcomes described above, 342 (94%) were solicited local, systemic, or laboratory events, 335 (92%) were mild, and 263 (72%) local to the injection site (Supplementary Table S4). The most common adverse events were discomfort and redness in the arm, discomfort in the leg, and blistering at the site of i.d. injection. Of 57 blistering events, 15 were moderate in grade, making this the commonest moderate adverse event observed in all groups, most frequent after the last immunization (Supplementary Tables S5 and S6).

### Concurrent i.d. and i.m. DNA vaccination with EP enhances antibody response to HIV-1 gp140 pre- and post-boosting with recombinant protein

**Figure 2.**
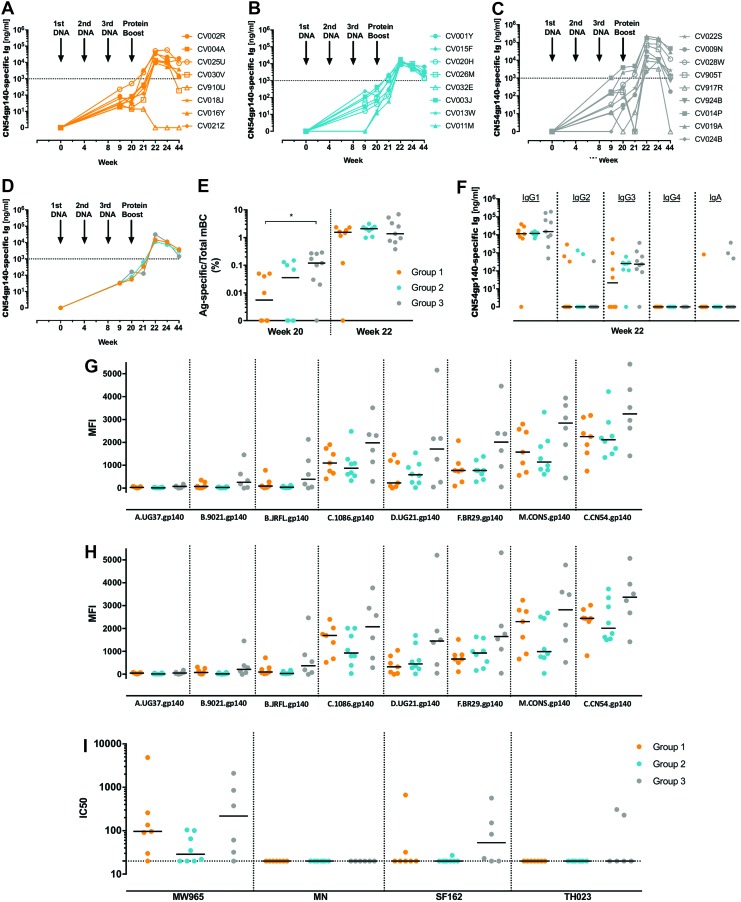
Antigen-specific humoral responses in human serum samples following DNA prime-protein boost vaccination regimens. Participants received 3 × priming immunizations of DNA-C *CN54ENV* at weeks 0, 4, and 8 by **(A)** i.d._EP_ + i.m. (*orange*), **(B)** i.d. + i.m._EP_ (*light blue*), and **(C)** i.d._EP_ + i.m._EP_ (*gray*) administration, followed by a common i.d. booster injection of recombinant CN54gp140 at week 20. Individual responses are shown in **(A–C)** and median responses in **(D)**. Percentage of Ag-specific memory B cells (mBC) per total immunoglobulin G (IgG)-secreting mBC as shown in **(E)** following DNA priming (week 20) and protein boost (week 22). mBC responses were significantly higher in i.d._EP_ + i.m._EP_ (group 3) relative to i.d._EP_ + i.m. at week 20 (*p* = 0.041). CN54gp140-specific IgG1-4 and IgA responses expressed as median are shown in **(F)**. The ability of vaccine-induced antibodies isolated at week 22 recognizing different HIV gp140 proteins to bind dimeric **(G)** FcγRIIIa or (**H**) FcγRIIa was assessed in a customized multiplex dimer assay. Serum neutralizing antibody titers, analyzed by TZM-bl assay **(I)**, data expressed as ID50 against a variety of viruses at week 22, 2 weeks after the protein boost. Statistical analysis was performed using a Kruskal–Wallis test with Dunn's correction for multiple comparisons. i.d._EP_ + i.m., received i.d. immunization with electroporation (EP) combined with intramuscular (i.m.) immunization; i.d. + i.m._EP_, received i.d. immunization combined with i.m. immunization with EP; i.d._EP_ + i.m._EP_, received i.d. immunization with EP combined with i.m. immunization with EP. i.m., intramuscular. Color images available online at www.liebertpub.com/hum

Antibody responses were determined 1 week post final DNA vaccination (week 9). All individuals in i.d._EP_ + i.m. (group1) seroconverted post third DNA vaccination (8/8; 100%). In i.d. + i.m._EP_ (group 2), 6/8 (75%) seroconverted by week 9, rising to 8/8 (100%) at the time of protein immunization. In i.d._EP_ + i.m._EP_, 8/9 (88.8%) individuals seroconverted by week 9, with an additional seroconversion detected on the day of protein boost (group 3; Fig. 2A–C). This late responder (CV024B) was the one participant where the TDS-ID device failed to deliver the first i.d. doses of the vaccine. Interestingly, for the majority of individuals across the groups, antibody responses continued to rise between weeks 9 and 20 (19/25; 76%). mBC ELISpot responses were concordant with seroconversion. Although there was no statistical difference between groups in the median antibody binding response at week 9 post third DNA immunization, mBC responses were higher in i.d._EP_ + i.m._EP_ (group 3) relative to i.d._EP_ + i.m. (group 1; Fig. 2E; *p* = 0.041). Nevertheless, all groups had similar mBC responses on protein boosting.

Following protein boost, participants from i.d._EP_ + i.m. (group 1) had a median antibody response of 13,934 ng/mL (Q1–Q3: 9,884–38,050 ng/mL; Fig. 2A and D) at 2 weeks (week 22) post protein vaccination (primary endpoint). For the participants followed out to week 44, this dropped to 2,751 ng/mL (Q1–Q3: 461–13,225 ng/mL). One participant in this group chose not to receive a protein boost (CV910U) but was followed out to study end, where the antibody responses had returned to baseline. A median response of 11,292 ng/mL (Q1–Q3: 9,608–15,568 ng/mL) 2 weeks post protein boost was seen in i.d. + i.m._EP_ (group 2). For those participants followed out to week 44, this fell to 3,012 ng/mL (Q1–Q3: 1,425–3,720 ng/mL; Fig. 2B and D). The median antibody response at 2 weeks post boost in i.d._EP_ + i.m._EP_ (group3) was 31,418 ng/mL (Q1–Q3: 6,304–128,259 ng/mL). For those participants in group 3 followed out to week 44, the median antibody response fell to 1,444 ng/mL (Q1–Q3: 476–20,634 ng/mL; Fig. 2C and D). When assessed by group, i.d._EP_ + i.m._EP_ (group 3) displayed a trend for earlier responses to the protein boost and elevated peak antibody response at the primary endpoint (week 22) when compared to groups 1 and 2 (Fig. 2A–C). However, this was not statistically significant. Post protein boost, mBC responses were seen in all individuals, except for the one individual in group 1 who declined their protein boost. Although there was no statistical difference between the three groups, the highest mean response was in i.d._EP_ + i.m._EP_ (group 3).

To understand the functionality of the induced response further, the induced antibody isotype profile was then determined. There was a predominant IgG1 response, with some sporadic induction of IgG2 and IgA (Fig. 2F). Interestingly, IgG3 responses, although low, were most reproducibly observed in groups 2 and 3 (7/8 and 8/9, respectively) while less frequent for group 1 (4/8). Subsequently, differences in the ability of vaccine-induced serum antibodies to interact with dimeric Fc receptors FcγRIIIa and/or FcγRIIa after binding to various HIV-1 clade Env proteins were investigated as an indirect readout for potential antibody dependent cellular cytotoxicity (ADCC) and antibody dependent cellular phagocytosis (ADCP).^30,31^ Env-specific antibodies with capacity to bind FcR dimers were induced by the different prime-boost vaccine regimens. Overall, the fingerprint of Fc-receptor binding, associated with different HIV-1 clades, was similar in pattern across the three groups. However, there was a trend for higher median response for both Env-specific FcγRIIIa and FcγRIIa binding antibodies in i.d._EP_ + i.m._EP_ (group 3; Fig. 2G and H). In all groups, homologous, group M consensus, clade C, F, and D binding vaccine-induced antibodies were detected by the dimeric FcγRIIIa and FcγRIIa, while vaccine-induced antibodies specific to clade B were low or absent, and clade A did not bind to dimeric FcγRIIIa and FcγRIIa. Low levels of neutralizing antibody were observed at week 22 against the closely matched clade C tier 1 isolate 93MW965, in line with previous observations using this envelope construct^28,29,33^ (Fig. 2I).

Cervical and rectal mucosal IgG and IgA specific antibody levels were also determined 2 weeks (week 22) post protein vaccination (primary endpoint). No rectal IgA was detected in any of the participants, with rectal IgG being detected at low levels in only one participant from group 1 and group 3 (35 ng/mL and 52 ng/mL, respectively). Each of the vaccination groups contained two females. Therefore, cervical antibodies were determined for these participants. Cervical IgA was not detected in women from group 2 or group 3, with one participant in group 1 showing negligible antibody levels of 13 ng/mL. Cervical IgG responses were detected in all women, irrespective of vaccination scheme, with median values of 104 ng/mL (group 1), 57 ng/mL (group 2), and 1,871 ng/mL (group 3; Supplementary Fig. S1).

### EP modulates the response profile of induced CD4 T cells in response to concurrent i.d. and i.m. DNA vaccination prior to boosting with recombinant protein

**Figure 3.**
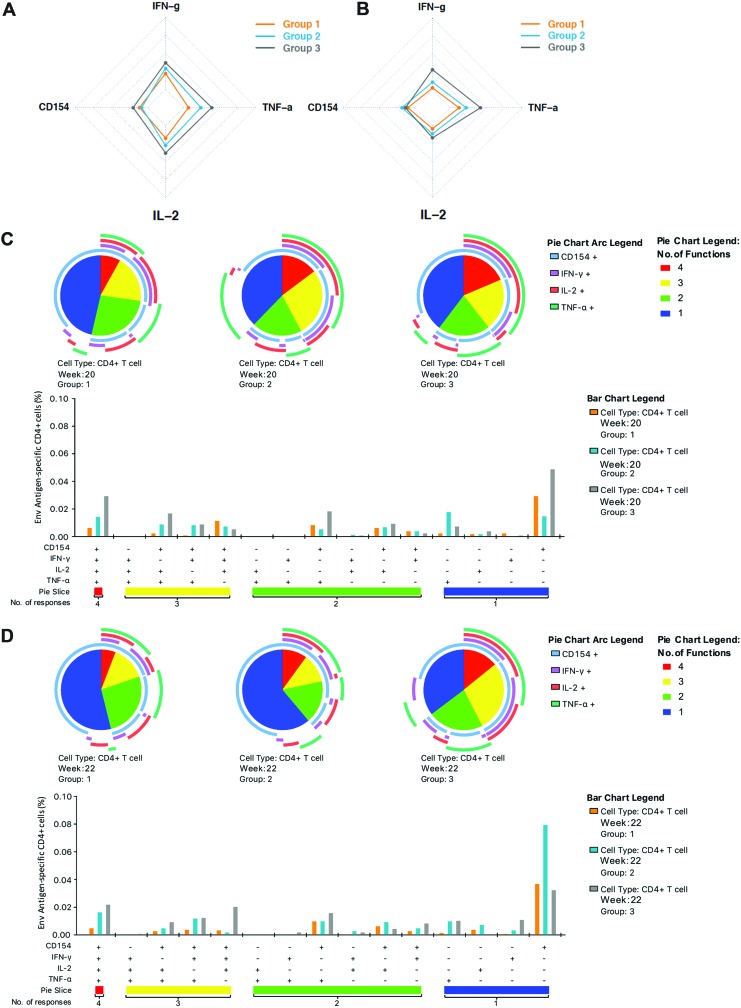
Intracellular cytokine staining (ICS) CD4 T-cell analysis. Baseline percentages have been subtracted from the week 20 and week 22 (primary endpoint) values **(A–D)**. Radar plots **(A** and **B)** demonstrate the mean T-cell response for each vaccination group according to cytokine expression levels as a marker of function following **(A)** DNA priming (week 20) and **(B)** protein boost (week 22). Pie charts compare the average functionalities of CD4+ T cells according to the three groups following DNA priming **(C)** and protein boost **(D)**. Polyfunctionality was defined as the concurrent expression of three or more functions. Pie slices denote proportions of CD4+ T cells co-expressing four (*red*), three (*yellow*), two (*green*), and one function (*blue*). Pie arcs represent proportions of the CD4+ T-cell response expressing CD154 (*light blue arcs*), interferon (IFN)-γ (*purple arcs*), interleukin (IL)-2 (*red arcs*), and tumor necrosis factor (TNF)-α (*light green arcs*). Bar graphs depict concurrent expression of CD154, IFN-γ, IL-2, and TNF-α within CD4+ T cells following stimulation with CN54 peptides. The *x*-axis represents positive (+) and negative (–) responses within each combination of CD154, IFN-γ, IL-2, and TNF-α functional subset. The *y*-axis represents the percentage of antigen-specific CD4+ T cells contributing a given functional subset. *Bars* indicate mean values. Statistical analysis was performed using a Kruskal–Wallis test with Dunn's correction for multiple comparisons. Color images available online at www.liebertpub.com/hum

Next, the response profile of the induced CD4 cellular immune response was assessed by ICS as a marker of CD4 T-cell help. Analysis of the total number of responders to any of the measured parameters following DNA vaccination (week 20) revealed a nonsignificant trend for fewer responders (5/8; 62.5%) in i.d._EP_ + i.m. (group 1) compared to 7/8 (87.5%) in i.d. + i.m._EP_ (group 2), and 7/9 (77.8%) in i.d._EP_ + i.m._EP_ (group 3). This trend was not changed upon protein boosting (week 22), whereby there were 4/8 (50%) responders in i.d._EP_ + i.m. (group 1) compared to 6/8 (75%) in i.d. + i.m._EP_ (group 2), and 7/9 (77.8%) in i.d._EP_ + i.m._EP_ (group 3). When analyzing the profile of responding CD4 T-cell populations following DNA vaccination (Fig. 3A), the magnitude of the induced TNF-α response was lower in i.d._EP_ + i.m. (group 1) compared to i.d. + i.m._EP_ (group 2; *p* = 0.048, chop-lump test^34^). Responses in i.d._EP_ + i.m._EP_ (group3) across all four markers (CD154, IFN-γ, TNF-α, and IL-2) were greater in magnitude than either i.d._EP_ + i.m. (group 1) or i.d. + i.m._EP_ (group 2) following DNA vaccination, although this did not reach statistical significance, except for TNF-α responses that were greater than those in group 1 (group 3; *p* = 0.041, chop-lump test). Furthermore, there was a trend for i.d._EP_ + i.m._EP_ (group 3) to have a higher fraction of polyfunctional cells expressing all four markers (Fig. 3C) compared to the other two groups. There was little change in the response rate and overall response profile following protein boost. However, there was a trend for increased TNF-α and INF-γ expression in i.d._EP_ + i.m._EP_ (group3; Fig. 3B) and higher levels of polyfunctionality (≥3 parameters) relative to the other two groups (Fig. 3D). Conversely, levels of single CD154-positive cells were increased in i.d._EP_ + i.m. (group 2) relative to groups 1 and 3 (Fig. 3D).

### CD8 T-cell responses are also differentially modulated by concurrent i.d. and i.m. DNA vaccination prior to boosting

**Figure 4.**
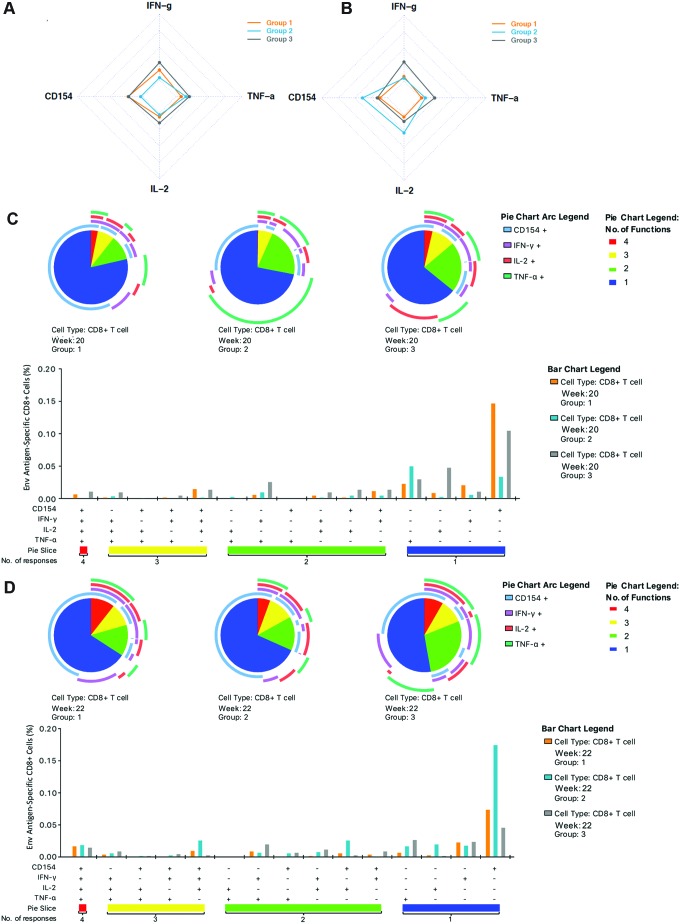
ICS CD8 T-cell analysis. Baseline percentages have been subtracted from the week 20 and week 22 (primary endpoint) values **(A–D)**. Radar plots **(A** and **B)** demonstrate the mean T-cell response for each vaccination group according to cytokine expression levels as a marker of function following **(A)** DNA priming (week 20) and **(B)** protein boost (week 22). Pie charts compare the average functionalities of CD8+ T cells according to the three groups following DNA priming **(C)** and protein boost **(D)**. Polyfunctionality was defined as the concurrent expression of three or more functions. Pie slices denote proportions of CD8+ T cells co-expressing four (*red*), three (*yellow*), two (*green*), and one function (*blue*). Pie arcs represent proportions of the CD8+ T-cell response expressing CD154 (*light blue arcs*), IFN-γ (*purple arcs*), IL-2 (*red arcs*), and TNF-α (*light green arcs*). Bar graphs depict concurrent expression of CD154, IFN-γ, IL-2, and TNF-α within CD8+ T cells following stimulation with CN54 peptides. The *x*-axis represents positive (+) and negative (–) responses within each combination of CD154, IFN-γ, IL-2, and TNF-α functional subset. The *y*-axis represents the percentage of antigen-specific CD8+ T cells contributing a given functional subset. Bars indicate mean values. Statistical analysis was performed using a Kruskal–Wallis Test with Dunn's correction for multiple comparisons. Color images available online at www.liebertpub.com/hum

While the primary focus of this study was on factors influencing humoral responses, to assess the wider implications of concurrent i.d. and i.m. DNA vaccination, the study also sought to determine the impact on CD8 responses. ICS analysis of the total number of CD8+ T-cell responders following DNA vaccination (week 20) revealed higher numbers in the two groups receiving i.d. EP, where i.d._EP_ + i.m. (group 1) and i.d._EP_ + i.m._EP_ (group 3) had 6/8 (75%) and 6/9 (66.7%) responders, respectively, while there were only two (25%) responders in i.d. + i.m._EP_ (group 2) who did not receive i.d. EP. The protein boost did not change the number of responders in i.d._EP_ + i.m._EP_ (group 3), but there was a reduction the number of responders in i.d._EP_ + i.m. (group 1; from 6/8 to 4/8), while an additional responder was seen in i.d. + i.m._EP_ (group 2; 3/8 [37.5%]). Analysis of the induced response profile in the responding CD8 cells following DNA vaccination revealed an inherent bias toward CD154 responses in the two groups receiving i.d._EP_ (groups 1 and 3) relative to the two responders in i.d. + i.m._EP_ (group 2; Fig. 4A). Boosting with recombinant gp140 shifted the response in i.d. + i.m._EP_ (group 2) toward a more CD154 dominated profile (Fig. 4B and D) with stronger IL-2 responses. Unlike CD4 T-cell responses, polyfunctional analysis revealed that the greatest proportion of CD8 T cells responding post DNA vaccination were single positive, with very few of the cells showing high poly-functionality associated with expression of three or four of the markers (Fig. 4C). As expected for exogenous protein immunogens, there was little change in the response profile following the boost (Fig. 4D).

### EP modulates IFN-γ T-cell ELISpot responses in healthy volunteers receiving concurrent i.d. and i.m. DNA vaccination prior to boosting with recombinant protein

**Figure 5.**
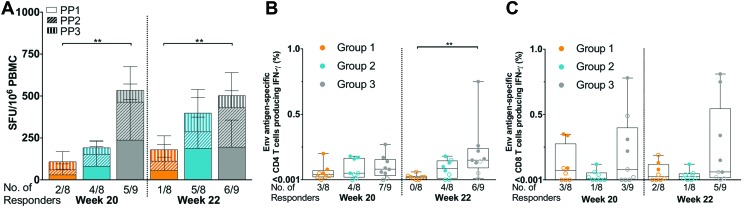
T-cell IFN-γ enzyme-linked immunospot responses following DNA priming (week 20) and protein boost (week 22), the primary endpoint **(A)**. All responses expressed as spot-forming units (SFU) per million peripheral blood mononuclear cells (PBMC), with background subtracted (SFU/10^6^ PBMC). Mean values ±95% confidence intervals are shown. *Shading* represents responses to the three peptide pools (PP). Antigen-specific CD4 **(B)** and CD8 **(C)** T cells producing IFN-γ at week 20 and week 22 as measured by ICS. The *boxes* show the median ± interquartile ranges and min/max values. Statistical analysis was performed using a Kruskal–Wallis Test with Dunn's correction for multiple comparisons. Color images available online at www.liebertpub.com/hum

To facilitate comparison across vaccine studies, the differential impact of the individual DNA immunization schedules on the induced cellular responses was also assessed by IFN-γ T-cell ELISpot. Positive IFN-γ responses 12 weeks post third DNA vaccination (week 20) were seen in 2/8 (25%) participants who received i.d._EP_ + i.m. (group 1), 4/8 (50%) who received i.d. + i.m._EP_ (group 2), and 5/9 (55.6%) who received i.d._EP_ + i.m._EP_ (group 3) vaccination (Fig. 5A). While there was no statistical difference in the number of responders between groups (chi-square test), the difference in magnitude of the IFN-γ response was statistically significant between i.d._EP_ + i.m._EP_ (group 3) and i.d._EP_ + i.m. (group 1; *p* = 0.001). Within i.d._EP_ + i.m._EP_, the greatest magnitude of IFN-γ response was to peptide pools 1 and 2, with mean values of 238 SFU/10^6^ PBMC (95% CI 0–480 SFU/10^6^ PBMC) and 227 SFU/10^6^ PBMC (95% CI 18–437 SFU/10^6^ PBMC) at week 20, respectively (Supplementary Fig. S2C). The subsequent protein boost was associated with a marginal change in response rate at the primary endpoint (week 22, 2 weeks following protein boost), where i.d._EP_ + i.m. (group 1) dropped to 1/8 (12.5%) participants and i.d._EP_ + i.m. (group 2) and i.d._EP_ + i.m._EP_ (group 3) increased to 5/8 (62.5%) and 6/9 (66.7%), respectively (Fig. 5A). There was a statistically significant difference between i.d._EP_ + i.m._EP_ (group 3) and i.d. + i.m._EP_ (group 1; *p* = 0.002). Statistical differences in the magnitude of response across all peptide pools when compared to background were observed for i.d. + i.m._EP_ (group 2) and i.d._EP_ + i.m._EP_ (group 3) but not for i.d._EP_ + i.m. (group 1; Supplementary Fig. S2A–C).

A similar trend was observed when IFN-γ responses were assessed by ICS, with the hierarchy in the number of responders being i.d._EP_ + i.m._EP_ > i.d. + i.m._EP_ > i.d._EP_ + i.m. Positive CD4 IFN-γ responses following DNA vaccination (week 20) were seen in 3/8 (37.5%) participants who received i.d._EP_ + i.m. (group 1), 4/8 (50%) who received i.d. + i.m._EP_ (group 2), and 7/9 (77.8%) who received i.d._EP_ + i.m._EP_ (group 3) vaccination (Fig. 5B). Subsequent to the protein boost, response rates decreased for i.d._EP_ + i.m. (group 1) to 0/8, remained constant for i.d. + i.m._EP_ (group 2), and decreased for i.d._EP_ + i.m._EP_ (group 3) to 6/9 (66.7%; week 22). Differences in the magnitude of the CD4 T-cell IFN-γ response by ICS were significant between i.d._EP_ + i.m._EP_ (group 3) and i.d._EP_ + i.m. (group 1) post protein boost (*p* = 0.006). There were fewer positive CD8 IFN-γ responses compared to CD4 responses, although interestingly these were lowest in i.d. + i.m._EP_ (group 2), suggestive of an association with i.d._EP_ and the induction of CD8 IFN-γ responses (Fig. 5C).

### Antibody functionality correlates to the magnitude of antigen-specific IgG but not CMI, regardless of vaccine regime

Finally, the data were analyzed to determine any association between the magnitude of binding antibody and any of the measured response parameters. There was no correlation between the magnitude of specific IgG and any of the cellular responses at week 22, both gamma interferon ELISpot or CD4/CD8 ICS (Supplementary Fig. S3). As anticipated, there was a strong correlation between total antigen-specific IgG and the predominant antigen-specific IgG1 response (*r* = 0.89) but no correlation with other isotypes. The magnitude of antigen-specific binding IgG also correlated with neutralization titer (*r* = 0.77 for 93MW965) and Fc-dimer receptor binding (FcγRIIa and FcγRIIIa; Supplementary Figures 3 and 4).

## Discussion

This study sought to develop a DNA priming strategy capable of reproducibly inducing detectable B-cell responses, evident before boosting with recombinant protein. The rationale behind this approach was to select conditions that would facilitate the use of DNA vaccines to evaluate a broad range of immunogens used in series or as a cocktail to focus humoral immune responses to recognize rarely induced broadly neutralizing epitopes^6^ prior to amplification with a conventional recombinant protein boost. However, while the ability of DNA to prime CD4 T-cell responses is well established, the extent to which DNA vaccination is able to prime antigen specific B cells directly is less clear. Indeed, a review of previous HIV vaccines studies performed by the HIV vaccine Trials Network suggests that antibody responses are rarely elicited by DNA priming in the absence of EP.^9^

To the best of the authors' knowledge, this is the first human study to determine the impact of DNA priming vaccination by combined i.d. and i.m. administration followed by i.d. boosting with recombinant protein. In general, the different modes of delivery (i.d._EP_ + i.m., i.d. + i.m._EP_, and i.d._EP_ + i.m._EP_) were well tolerated, with no serious adverse events reported during the trial. Encouragingly, 100% seroconversion was seen prior to the protein boost across all three DNA priming regimes. Although there was no statistical difference in the median binding antibodies, the highest mBC responses were observed in i.d._EP_ + i.m._EP_. Furthermore, following the protein boost, i.d._EP_ + i.m._EP_ (group 3) displayed a trend for earlier responses and elevated peak antibody responses at the primary endpoint compared to groups 1 and 2. Larger studies will be needed to determine if these differences are statistically significant. It should be noted that i.d. and i.m. immunizations were administered to different limbs (arm and leg respectively) that drain to different lymph nodes. Whether this anatomical separation of the two injections is necessary for the observed effects warrants further study. The dose of DNA delivered i.d. was also considerably lower (600 μg) than the i.m. dose (2 mg). Therefore, it cannot be excluded that administration of two concurrent i.d./EP doses might be equivalent or greater than that of combined i.d./EP + i.m./EP with respect to antibody priming. A further limitation of this study is the lack of a control group that did not receive EP by either route of administration. However, previous studies showing the strong impact of EP on induced responses suggest this would have been inferior.^10–16^ There are no directly comparable human studies investigating combined i.d. + i.m. immunization to either i.d. or i.m. administered in the context of EP. The closest matched study is that of Vasan *et al.*^16^ comparing i.m. administration of DNA-based candidate HIV-1 vaccine expressing Clade C/B′ *env*, *gag*, *pol*, *nef,* and *tat* genes with and without EP delivered by the same device as used in this study. In this previous study, only one participant displayed a transient very weak antibody response to gp120. A second study exploring the use of ID DNA administration for a multivalent vaccine encoding Env, Gag, Pol, with and without EP using a different device (Dermavax), failed to show any benefit from EP and no evidence of seroconversion to the DNA priming phase.^17^

However, the responses to multigenic vaccines are likely to be different to those encoding a single HIV-1 Env sequence. A parallel ongoing study (DNAVAC) using the same DNA vaccine has failed to produce significant seroconversion following three immunizations with either i.d._EP_ or i.m._EP_ (pers. commun.). These data suggest that combined i.d. and i.m. immunization in the context of EP is superior to either i.d. or i.m. immunization alone.

It should be noted that two previous clinical studies have reported seroconversion to HIV-1 envelope following DNA priming in the absence of EP. The first of these assessed responses following three injections of a six-plasmid HIV-1 DNA vaccine encoding gp145 of subtype A, B, C, and Env and subtype B Gag, Pol, and Nef administered by a needle-free Biojector^®^2000.^7^ This study reported a 71% seroconversion rate, with a median antibody titer of 800 to Env (A, C, B). Although the Biojector administration was targeted to the lateral deltoid muscle, the ballistic nature of administration likely distributed DNA across both dermal and muscle tissue. A second study utilized a six-plasmid HIV-1 DNA vaccine encoding codon-optimized gp120 Env from subtypes, A, B( × 2), C, and E, and a subtype C Gag.^8^ When administered at a high dose (7.2 mg at each immunization) via i.m. injection divided between two sites in the absence of EP, 4/6 (66.7%) volunteers showed detectable Env-specific IgG, with median binding titers of 1,600. The higher dose, greater intrinsic immunogenicity of gp120 monomers over trimeric gp140, and differences in expression plasmids likely account for higher seroconversion rates when compared to other studies. It is certainly possible that this latter study did not involve the use of EP and might also be effective for priming responses to subsequent protein boost. Nevertheless, neither of these two previous studies was able to match the 100% seroconversion following the combined i.d./i.m. DNA priming observed in this study. Further work is required to determine whether such a difference provides significant justification for the more complicated delivery procedure. Interestingly, a prototype device for simultaneous EP-enhanced DNA vaccine delivery to both skin and muscle is under development, offering the potential to simplify the approach.^35^

In the current study, the magnitude of induced humoral responses following a single i.d. boost with recombinant CN54 gp140 in the absence of adjuvant was similar to that previously observed following two immunizations with the same recombinant protein (CN54 gp140) delivered with the TLR adjuvant GLA-AF^28^ or following different DNA-MVA priming regimes.^33^ The different DNA prime-protein boost regimes in this study induced a predominant IgG1 response with lower levels of IgG3. IgG2 responses were less evident than in previous studies using recombinant CN54 gp140 adjuvanted in GLA-AF,^28^ suggesting the DNA priming phase promoted a more Th1 dominant response. In line with previous studies CN54 gp140 induced low neutralizing antibody responses against a closely matched clade C tier 1 isolate MW965 with minimal reactivity against clade B MN and SF162 and clade A/E recombinant TH023.^28,29,33^ However, vaccine-induced serum antibodies were seen to interact with dimeric Fc receptors FcγRIIIa and/or FcγRIIa, surrogates of both ADCC and ADCP, respectively.^30,31^ In particular clade C, F, and D binding vaccine-induced antibodies were detected by the dimeric FcγRIIIa and FcγRIIa. Similar to the neutralization profile, vaccine-induced antibodies specific to clade A and B were either absent or too low to crosslink dimeric FcγRIIIa and FcγRIIa. These responses mirror previous observations seen when using recombinant protein alone.^28^ The potential importance of ADCC has been highlighted by the RV144 trial in which robust HIV-specific ADCC responses were linked to the observed partial protection.^36–38^ Data using the CN54 gp140 Env suggest potential ADCC and/or ADCP would be restricted to clades C, F, and D.

Intramuscular EP appeared to have a positive impact on the response frequency of Env-specific CD4 responses elicited after three DNA immunizations with i.d. + i.m._EP_ and i.d._EP_ + i.m._EP_, showing 97.5% and 77.8% responses, respectively, compared to 62.5% for i.d._EP_ + i.m. Although not statistically relevant, this positive association fits earlier observations that i.m._EP_ preferentially induces CD4 T-cell responses.^16^ Furthermore, CD4 responses in i.d._EP_ + i.m._EP_ across all four markers (CD154, IFN-γ, TNF-α, and IL-2) were greater in magnitude and had a higher fraction of polyfunctional cells (≥3 markers) than the other two groups. By contrast, i.d._EP_ appeared to have a positive impact on the response frequency of Env-specific CD8 responses, where i.d._EP_ + i.m. and i.d._EP_ + i.m._EP_ had 75% and 66.7% response rates, respectively, compared to 25% in i.d. + i.m._EP_ group. Preferential induction of CD8 T cells responses by DNA vaccination of the skin concords with previous clinical studies using a T cell-based DNA vaccine.^27^ Interestingly, when response rate was determined by IFN-γ T-cell ELISpot, often assumed to be a marker of CTL response, hierarchy in the number of responders was i.d._EP_ + i.m._EP_ > i.d. + i.m._EP_ > i.d._EP_ + i.m., more reflective of CD4 T-cell responses. However, further analysis by ICS demonstrated a predominance of IFN-γ-positive CD4 T cells relative to CD8 T cells. This is in line with previous studies by Vasan *et al.* showing a predominant IFN-γ CD4 T-cell response following i.m._EP_^16^ that were of a similar magnitude and frequency.

Correlation analysis failed to show an association between any of the measured cell-mediated response parameters and the magnitude of binding antibody. These data suggest that the measured cellular parameters have no impact on the magnitude of induced antibody and are not predictive of lymph node Tfh responses required to provide B-cell help. Future studies will be required to assess the induction of better biomarkers of Tfh responses in the systemic compartment and/or responding lymph nodes.^39^ Unsurprisingly, there was a strong correlation between total antigen-specific IgG and antigen-specific IgG1 responses (*r* = 0.89), with IgG1 being the predominant isotype response. The magnitude of antigen-specific binding IgG also correlated with neutralization titer (*r* = 0.77 for 93MW965) and Fc-dimer receptor binding (FcγRIIa and FcγRIIIa; Supplementary Fig. S4). Stronger correlations with FcγRIIa compared to FcγIIIa likely reflect the observed subclass distribution of IgG1, 2, and 3 levels. IgG1 and IgG3 bind well to both FcγRIIa and IIIa, while IgG2 only binds well to FcγRIIa.

Collectively, the data presented here support the concept for the use of concurrent i.d. and i.m. DNA priming vaccination combinations as mediators of robust T- and B-cell responses, with protein boost immunizations serving to amplify antibody levels efficiently. The study objective of identifying a DNA priming strategy capable of reproducibly inducing detectable antibody responses in the majority of subjects was met. While the three DNA priming strategies each induced 100% seroconversion, i.d._EP_ + i.m._EP_ performed best. Interestingly, responses for the majority of the participants were maintained or increased over the 12 weeks following the third DNA vaccination and the time of the protein boost. It would be interesting to know if additional concurrent DNA immunization might provide further gains in antibody titer. Molecular adjuvants were not used in order to differentiate clearly the impacts of the different components of the concurrent regimens. However, additional gains might be mediated by the future inclusion of molecular adjuvants in the DNA-priming phase with potential impact on avidity and neutralization breadth. In the current phase of development, a single DNA plasmid was used as a proof-of-concept study. The studies clearly demonstrate that the combined i.d._EP_ + i.m._EP_ protocol is capable of directly priming antigen-specific B cells. In future studies, it will be important to determine whether a series of DNA immunogens and/or combinations can drive B-cell responses toward rarely induced broadly neutralizing antibodies, with their potential amplification with a single protein boost. Such an approach has obvious attractions, given the relative savings in speed and cost of DNA manufacture compared to other vaccine platforms. In this respect, the DNA prime-protein boost approach has potential not only to accelerate the testing of candidate HIV-1 vaccines with the aim of elucidating immunogens capable of inducing broadly neutralizing antibody responses, but also to provide a new platform for the development of future vaccines against a wide range of existing or emerging pathogens.

## Supplementary Material

Supplemental data

Supplemental data

Supplemental data

Supplemental data

Supplemental data

Supplemental data

Supplemental data

Supplemental data

Supplemental data

Supplemental data
