# Supplementary material for: Combined Skin and Muscle DNA Priming Provides Enhanced Humoral Responses to a Human Immunodeficency Virus Type 1 Clade C Envelope Vaccine
Source: Hum Gene Ther. 2018 Oct 17;29(9):1011–28. doi: 10.1089/hum.2018.075 (PMC6214652; doi:10.1089/hum.2018.075)
Supplement: Supplemental data [file Supp_Table1.pdf]

Supplementary Table S1. *Trial inclusion and exclusion criteria*

Inclusion criteria	*Exclusion criteria*
Men and women aged between 18 and 50 years inclusive on the day of screening<br>Body mass index between 18 and 30 kg/m^2 inclusive<br>Willing and able to give informed written consent	Pregnant or lactating<br>Skin-fold measurement of upper thigh >40 mm<br>Unable to read and speak English to a fluency level adequate for the full comprehension of procedures required in participation and consent, or unlikely to comply with the protocol
At low risk of HIV and willing to remain so for the duration of study defined as:<br>• No history of injecting drug use in last 10 years<br>• No gonorrhea or syphilis infection in last 6 months<br>• No "high-risk" partner (*e.g.*, IDU, HIV positive) currently or in last 6 months<br>• No unprotected anal intercourse in last 6 months outside of relationship with regular HIV-negative partner<br>• No unprotected vaginal intercourse in last 6 months outside of relationship with regular known/presumed HIV-negative partner	Clinically relevant abnormality on history or examination including:<br>• History of grand-mal epilepsy, seizure disorder, or any history of prior seizure<br>• History of cardiac arrhythmia or palpitations<br>• History of syncope or fainting episodes within 1 year of enrolment<br>• Known hypersensitivity to any of the components in the vaccines or severe or multiple allergies to drugs or pharmaceutical agents<br>• Treatment with immunosuppressives in last 3 months (oral, inhaled, nasal, or injected)<br>• Surgical or traumatic metal implants at the sites of administration<br>• Current use of an electronic stimulation device
If heterosexually active female capable of becoming pregnant, must (in addition to using male condoms) agree to use hormonal contraception or abstain from 14 days before the first vaccination until 4 months after the last<br>If heterosexually active male, must agree to use condoms from 14 days prior to the first vaccination until 4 months after the last	Women using a intrauterine device for contraception or with a past history of toxic shock syndrome
Agree to abstain from blood donation for 3 months after the end of trial or longer if necessary	HIV-1 or -2 positive or indeterminate, HepBsAg-positive, HepC Ab-positive, or active syphilis
Available for follow-up for around 12 months from screening	Grade 1 or above abnormal laboratory parameters (including conjugated hyperbilirubinemia)
Willing to undergo HIV test and screen for genital infection screen	Previous severe reaction to vaccination or receipt of live attenuation vaccine in last 60 days or any other vaccine within 14 days of enrolment
Registered with a GP for at least the past 3 months	Receipt of an experimental vaccine containing HIV envelope components at any time in the past, or completion of another trial of a medicinal product within 30 days of enrolment
Entered and clearance obtained from the Over-volunteering Prevention System database	Receipt of blood products or immunoglobulin within 4 months of screening
