# Supplementary material for: Combined Skin and Muscle DNA Priming Provides Enhanced Humoral Responses to a Human Immunodeficency Virus Type 1 Clade C Envelope Vaccine
Source: Hum Gene Ther. 2018 Oct 17;29(9):1011–28. doi: 10.1089/hum.2018.075 (PMC6214652; doi:10.1089/hum.2018.075)
Supplement: Supplemental data [file Supp_Table2.pdf]

Supplementary Table S2. *Demographic characteristics of randomized participants* (N = 24)

	i.d./EP (n = *8)*	*i.m./EP* (n = *8)*	*i.m./i.d./EP* (n = *8)*	*All* (N = *24)*
Age (years)	31 (25–34)	27 (23–43)	22 (21–47)	27 (22–43)
Sex (male)	6 (75%)	6 (75%)	6 (75%)	18 (75%)
Ethnicity				
White British	6	5	6	17
White other	1	0	2	3
Other	1	3	0	4
Weight (kg)	82 (76–85)	62 (57–75)	73 (67–83)	75 (66–83)
BMI (kg/m^2)	24 (23–26)	21 (20–24)	25 (23–27)	24 (21–25)

Data shown are number (%) or median (interquartile range).
