# Supplementary material for: Combined Skin and Muscle DNA Priming Provides Enhanced Humoral Responses to a Human Immunodeficency Virus Type 1 Clade C Envelope Vaccine
Source: Hum Gene Ther. 2018 Oct 17;29(9):1011–28. doi: 10.1089/hum.2018.075 (PMC6214652; doi:10.1089/hum.2018.075)
Supplement: Supplemental data [file Supp_Table3.pdf]

Supplementary Table S3. *Demographic characteristics of non-randomized participants*

	i.d./EP (n = 1)	*i.m./EP* (n = 0)	*i.m./i.d./EP* (n = 3)	*All* (N = 4)
Age (years)	23		35 (27,48)	31 (27,35)
Sex (male)	1 (100%)	0	2 (67%)	3 (75%)
Ethnicity				
White British	1		1	2
White other			1	1
Other			1	1
