# Supplementary material for: Combined Skin and Muscle DNA Priming Provides Enhanced Humoral Responses to a Human Immunodeficency Virus Type 1 Clade C Envelope Vaccine
Source: Hum Gene Ther. 2018 Oct 17;29(9):1011–28. doi: 10.1089/hum.2018.075 (PMC6214652; doi:10.1089/hum.2018.075)
Supplement: Supplemental data [file Supp_Table4.pdf]

Supplementary Table S4. *Summary of solicited local, systemic, or laboratory adverse events, any event leading to treatment discontinuation, and any unsolicited adverse event starting within 7 days of immunization*

	i.d./EP (n = *8)*	*i.m./EP* (n = *8)*	*i.m./i.d./EP* (n = *8)*	*Total* (N = *24)*
Participants with ≥1 event	7 (88%)	8 (100%)	8 (100%)	23 (96%)
Events per participant				
0	1	0	0	1
1–4	0	0	1	1
5–9	1	1	1	3
10–19	3	6	3	12
≥20	3	1	3	7
Total number of events	120 (100%)	115 (100%)	130 (100%)	365 (100%)
Type of event				
Solicited local	80 (67%)	91 (79%)	92 (71%)	263 (72%)
Solicited systemic	25 (21%)	13 (11%)	18 (14%)	56 (15%)
Systemic laboratory	4 (3%)	7 (6%)	12 (9%)	23 (6%)
Unsolicited	11 (9%)	4 (3%)	8 (6%)	23 (6%)
Grade of event				
1	112 (93%)	104 (90%)	119 (92%)	335 (92%)
2	7 (6%)	10 (9%)	11 (8%)	28 (8%)
3	1[a] (1%)	1[b] (1%)	0 (0%)	2 (1%)
4	0 (0%)	0 (0%)	0 (0%)	0 (0%)
Relationship to vaccine				
Definite	61 (80%)	68 (93%)	64 (81%)	193 (85%)
Probable	3 (4%)	2 (3%)	2 (3%)	7 (3%)
Possible	3 (4%)	0 (0%)	4 (5%)	7 (3%)
Unlikely	4 (5%)	0 (0%)	1 (1%)	5 (2%)
None	5 (7%)	3 (4%)	8 (10%)	16 (7%)
Missing[c]	44	42	51	137

No safety events occurred that led to discontinuation of vaccine.
[a]Arm pain.
[b]Elevated aspartate transaminase.
[c]Relatedness not recorded for solicited events collected on diary cards.
