# Supplementary material for: Combined Skin and Muscle DNA Priming Provides Enhanced Humoral Responses to a Human Immunodeficency Virus Type 1 Clade C Envelope Vaccine
Source: Hum Gene Ther. 2018 Oct 17;29(9):1011–28. doi: 10.1089/hum.2018.075 (PMC6214652; doi:10.1089/hum.2018.075)
Supplement: Supplemental data [file Supp_Table5.pdf]

Supplementary Table S5. *Local solicited adverse events—arm*

Symptom	Maximum grade	*i.d./EP*				*i.m./EP*				*i.m./i.d./EP*			
		1, n = *8*[a]	*2,* n = *7*[a]	*3,* n = *7*[a]	*4,* n = *7*[a]	*1,* n = *8*[a]	*2,* n = *8*[a]	*3,* n = *8*[a]	*4,* n = *8*[a]	*1,* n = *8*[a]	*2,* n = *7*[a]	*3,* n = *7*[a]	*4,* n = *6*[a]
Discomfort	1	6	5	5	6	1	2	1	4	2	3	2	6
	2	0	0	0	0	0	0	0	1	0	0	0	0
	3	0	0	1	0	0	0	0	0	0	0	0	0
	4	0	0	0	0	0	0	0	0	0	0	0	0
Redness	1	3	4	4	6	4	3	4	7	3	4	4	6
	2	0	0	0	0	0	0	0	0	0	0	0	0
	3	0	0	0	0	0	0	0	0	0	0	0	0
	4	0	0	0	0	0	0	0	0	0	0	0	0
Swelling (soft)	1	1	0	2	2	1	1	3	0	0	2	1	2
	2	0	0	0	0	0	0	0	0	0	0	0	0
	3	0	0	0	0	0	0	0	0	0	0	0	0
	4	0	0	0	0	0	0	0	0	0	0	0	0
Induration (hard)	1	2	1	2	3	2	2	2	6	2	3	2	5
	2	0	0	0	1	0	0	0	0	0	0	0	0
	3	0	0	0	0	0	0	0	0	0	0	0	0
	4	0	0	0	0	0	0	0	0	0	0	0	0
Blisters	1	4	2	2	3	4	8	4	6	2	1	2	3
	2	0	0	0	3	3	0	0	2	0	2	2	3
	3	0	0	0	0	0	0	0	0	0	0	0	0
	4	0	0	0	0	0	0	0	0	0	0	0	0

[a]Vaccination number, *n* = number at risk.
