# Supplementary material for: Combined Skin and Muscle DNA Priming Provides Enhanced Humoral Responses to a Human Immunodeficency Virus Type 1 Clade C Envelope Vaccine
Source: Hum Gene Ther. 2018 Oct 17;29(9):1011–28. doi: 10.1089/hum.2018.075 (PMC6214652; doi:10.1089/hum.2018.075)
Supplement: Supplemental data [file Supp_Table6.pdf]

Supplementary Table S6. *Local solicited adverse events—leg*

		i.d./EP			*i.m./EP*			*i.d./i.m./EP*		
Symptom	*Maximum grade*	*1,* n = *8*[a]	*2,* n = *7*[a]	*3,* n = *7*[a]	*1,* n = *8*[a]	*2,* n = *8*[a]	*3,* n = *8*[a]	*1,* n = *8*[a]	*2,* n = *7*[a]	*3,* n = *6*[a]
Discomfort	1	3	1	2	8	6	3	7	5	7
	2	0	0	0	0	0	0	0	0	0
	3	0	0	0	0	0	0	0	0	0
	4	0	0	0	0	0	0	0	0	0
Redness	1	0	1	2	0	0	1	2	2	1
	2	0	0	0	0	0	0	0	0	0
	3	0	0	0	0	0	0	0	0	0
	4	0	0	0	0	0	0	0	0	0
Swelling (soft)	1	0	0	1	0	0	0	1	1	0
	2	0	0	0	0	0	0	0	0	0
	3	0	0	0	0	0	0	0	0	0
	4	0	0	0	0	0	0	0	0	0
Induration (hard)	1	0	0	1	1	1	0	2	1	1
	2	0	0	0	0	0	0	0	0	0
	3	0	0	0	0	0	0	0	0	0
	4	0	0	0	0	0	0	0	0	0
Blisters	1	0	0	1	0	0	0	0	0	0
	2	0	0	0	0	0	0	0	0	0
	3	0	0	0	0	0	0	0	0	0
	4	0	0	0	0	0	0	0	0	0

[a]Vaccination number, *n* = number at risk.
