# Supplementary material for: Combined Skin and Muscle DNA Priming Provides Enhanced Humoral Responses to a Human Immunodeficency Virus Type 1 Clade C Envelope Vaccine
Source: Hum Gene Ther. 2018 Oct 17;29(9):1011–28. doi: 10.1089/hum.2018.075 (PMC6214652; doi:10.1089/hum.2018.075)
Supplement: Supplemental data [file Supp_Fig1.pdf]

Supplementary Data

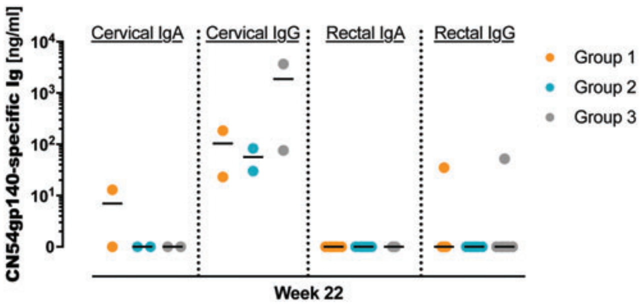

Supplementary Figure S1. Antigen-specific humoral responses in human mucosal secretions following DNA prime-boost vaccination regimens. Individual IgG and IgA responses within cervical and rectal secretions are shown (ng/mL) for participants post protein boost (week 22), the primary endpoint. i.d.$_{EP}$ + i.m. (*orange*, group 1), i.d. + i.m.$_{EP}$ (*light blue*, group 2), and i.d.$_{EP}$ + i.m.$_{EP}$ (*gray*, group 3). Bars represent median points.
