# Supplementary material for: Combined Skin and Muscle DNA Priming Provides Enhanced Humoral Responses to a Human Immunodeficency Virus Type 1 Clade C Envelope Vaccine
Source: Hum Gene Ther. 2018 Oct 17;29(9):1011–28. doi: 10.1089/hum.2018.075 (PMC6214652; doi:10.1089/hum.2018.075)
Supplement: Supplemental data [file Supp_Fig2.pdf]

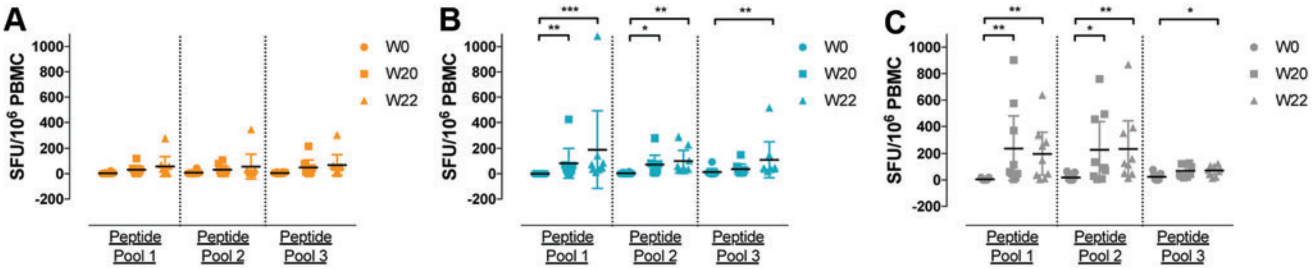

Supplementary Figure S2. T-cell IFN-γ ELISpot responses to the individual CN54 peptide pools at baseline (week 0, *circles*), following DNA priming (week 20, *squares*) and protein boost (week 22, *triangles*), the primary endpoint. i.d.$_{EP}$ + i.m. (*orange*, group 1) **(A)**, i.d. + i.m.$_{EP}$ (*light blue*, group 2) **(B)**, or i.d.$_{EP}$ + i.m.$_{EP}$ (*gray*, group 3) **(C)**. All responses are expressed as spot-forming units per million PBMC, with background subtracted (SFU/10^6 PBMC). Bars represent mean ± 95% CI. Statistical analysis was performed using Kruskal–Wallis Test with Dunn's correction for multiple comparisons.
