# Supplementary material for: Combined Skin and Muscle DNA Priming Provides Enhanced Humoral Responses to a Human Immunodeficency Virus Type 1 Clade C Envelope Vaccine
Source: Hum Gene Ther. 2018 Oct 17;29(9):1011–28. doi: 10.1089/hum.2018.075 (PMC6214652; doi:10.1089/hum.2018.075)
Supplement: Supplemental data [file Supp_Fig4.pdf]

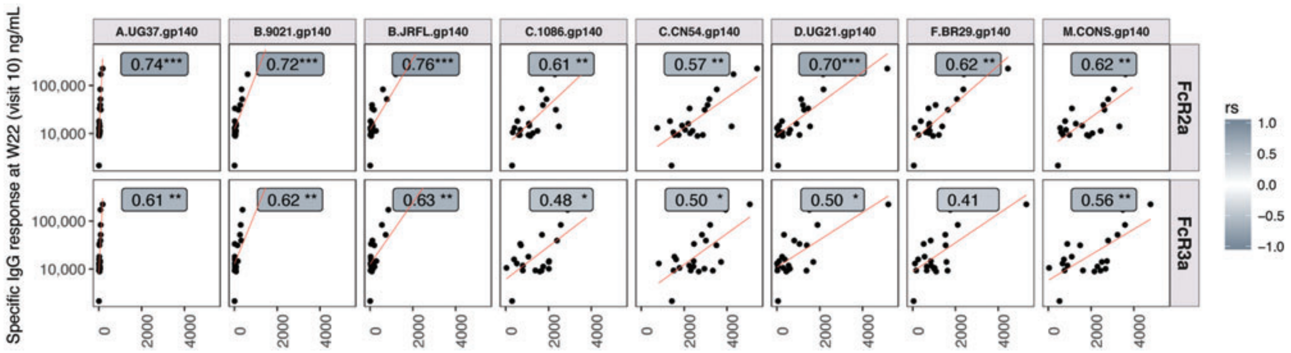

Supplementary Figure S4. Correlation analysis of antigen-specific serum IgG antibody responses at week 22, primary endpoint plotted against FcγR (2a/IIa and 3a/IIIa) dimer assay data, as shown in the scatter plots. Correlations were calculated by Spearman's rank correlation coefficient (r_S) and shown in *boxes*. The *box colors* reflect degree of correlation. The significance of the correlations is represented by asterisks: *$p<0.05$; **$p<0.01$; and ***$p<0.001$.
